# A Novel Five-Dimensional Three-Leaf Chaotic Attractor and Its Application in Image Encryption

**DOI:** 10.3390/e22020243

**Published:** 2020-02-21

**Authors:** Tao Wang, Liwen Song, Minghui Wang, Shiqiang Chen, Zhiben Zhuang

**Affiliations:** 1 College of Computer Science, Sichuan University, Chengdu 610000, China; 2000012@hbmzu.edu.cn (T.W.); wangminghui@scu.edu.cn (M.W.); 2 School of Advanced Materials and Mechatronic Engineering, Hubei Mizu University, Enshi 445000, China; 1997013@hbmzu.edu.cn; 3 School of Science, Hubei Minzu University, Enshi 445000, China; 201930081@hbmy.edu.cn

**Keywords:** five-dimensional chaotic system, digital image encryption, proportional block, convolution kernel

## Abstract

This paper presents a novel five-dimensional three-leaf chaotic attractor and its application in image encryption. First, a new five-dimensional three-leaf chaotic system is proposed. Some basic dynamics of the chaotic system were analyzed theoretically and numerically, such as the equilibrium point, dissipative, bifurcation diagram, plane phase diagram, and three-dimensional phase diagram. Simultaneously, an analog circuit was designed to implement the chaotic attractor. The circuit simulation experiment results were consistent with the numerical simulation experiment results. Second, a convolution kernel was used to process the five chaotic sequences, respectively, and the plaintext image matrix was divided according to the row and column proportions. Lastly, each of the divided plaintext images was scrambled with five chaotic sequences that were convolved to obtain the final encrypted image. The theoretical analysis and simulation results demonstrated that the key space of the algorithm was larger than 10^150^ that had strong key sensitivity. It effectively resisted the attacks of statistical analysis and gray value analysis, and had a good encryption effect on the encryption of digital images.

## 1. Introduction

In recent years, chaotic and hyperchaotic systems that can produce various types and are suitable for secure communication have become topics of great interest in the fields of physics, biomathematics, and information security [1]. Compared with the traditional encryption method, the complex structure and dynamic behavior of chaotic attractors have a better encryption effect for digital image encryption [2]. Therefore, it has become more important to construct chaotic attractors with multiple scrolls. In the process of encrypting digital images, the core purpose is to change the position of pixels and the size of pixel values. Therefore, experts and scholars have proposed many encryption algorithms such as using chaotic sequences to perform bit disturb of images [3,4], using the chaotic sequence and image pixel value for the XOR operation [5,6], and scrambling the pixel [7,8,9]. A nonlinear state feedback controller was proposed in Reference [10] based on the original three-dimensional (3D) autonomous chaotic system to construct a new four-dimensional hyperchaotic system. An image encryption algorithm based on five-dimensional hyper-chaos and bit-level disturbance was proposed in Reference [11]. Chaotic image encryption algorithms based on bit-level scrambling and dynamic DNA coding were proposed in Reference [12]. Liu et al. [13] proposed an image encryption algorithm for bit position chaos on the upper four bits. On the basis of arranging the diffusion structure, to improve safety and sensitivity, a chaotic image encryption algorithm based on a breadth-first search and dynamic diffusion was proposed in Reference [14]. The cryptosystem in Reference [15] uses a diffusion layer and then positions the image in layers instead of byte permutations to disturb the position of the image pixels. A new chaotic system with hidden attractors and a chaotic-based image encryption algorithm with a random number generator was proposed in Reference [16]. To reduce the processing time, Enayatifar et al. [17] performed simultaneous replacement and diffusion steps for any pixel.

Many chaotic-based image encryption algorithms have been inspired by Fridrich’s method. Therefore, this architecture has become most well-known [18]. However, security of an efficient image encryption method is a fundamental issue in an image encryption algorithm. Recent cryptanalytical studies have proven that some chaos-based algorithms are not adequately secure to resist against a common attack such as Reference [19].

In the proposed scheme, the new five-dimensional chaotic system can generate three-leaf chaotic attractors in multiple directions. Simultaneously, its dynamic characteristics are analyzed. In the process of encryption, to increase the key space, we perform convolution operations on five chaotic sequences. Second, we scale the image matrix proportionally and scramble the image matrix for each segment separately. Through these processes, the difficulty of the exhaustive attack is increased. All except for the exhaustive method based on the explicit plaintext ciphertext mapping, the method will be invalid and have high security.

The rest of the paper is organized as follows. In Section 2, the chaotic system model is given. In Section 3, circuit design and experimental results are described. In Section 4, related knowledge is introduced. The encryption scheme is described in Section 5. Simulation results and performance analyses are reported in Section 6. Lastly, the conclusions are drawn in Section 7.

## 2. New Five-Dimensional Chaotic System

In 1994, Sprott [20] summarized many 3D chaotic systems. After analyzing these 3D chaotic systems, we propose a new 3D chaotic system. The system equations are as follows.

(1) $$\left\{ \begin{array}{l} \dot{x}=ax+byz^{2}\\ \dot{y}=cx+dz^{2}\\ \dot{z}=e+fx \end{array}\right.$$

when *a* = −1, *b* = −3, *c* = 1, *d* = 1, *e* = 1, *f* = 1, system (1) is in a chaotic state.

Based on system (1), we introduce two controllers, *w*, *v*, and feedback *w* into the original controller *y*, feedback *v* into the original controller *w*, original controller *x* feedback into the new controller *v*, original controller *y* feedback into the new controller *w*, *v*, original controller *z* feedback to the new controller *w*, and feedback *v* to *w*. These six operations make the five controllers of this system interact with each other, which makes the relationship more complicated. The newly constructed five-dimensional chaotic system is as follows.

(2) $$\left\{ \begin{array}{l} \dot{x}=ax+byz^{2}\\ \dot{y}=x+z^{2}-wz\\ \dot{z}=1+x\\ \dot{w}=yz+cw+dv\\ \dot{v}=xy+ev \end{array}\right.$$

when parameters *a* = −1, *b* = −3, *c* = −3, *d* = 1.8, *e* = −5, the system is in a chaotic state.

### 2.1. Dissipative Analysis

Because of

(3) $$\Delta V=\frac{\partial \dot{x}}{\partial x}+\frac{\partial \dot{y}}{\partial y}+\frac{\partial \dot{z}}{\partial z}+\frac{\partial \dot{w}}{\partial w}+\frac{\partial \dot{v}}{\partial v}=a+c+e=-9<0$$

Therefore, system (2) is dissipative, and convergence is in an exponential form of *V*_0_*e*^−(*a*+*c*+*e*)*t*^. Clearly, the volume element *V*_0_ shrinks to the volume *V*_0_*e*^−(*a*+*c*+*e*)*t*^ at moment *t*. Now consider when t→∞. Each volume element that contains the system trajectory shrinks to 0 at an exponential rate *a* + *c* + *e* = −9.

### 2.2. Balance Point Analysis

Let *x* = *y* = *z* = *w* = *v* = 0, that is,

(4) $$\left\{ \begin{array}{l} ax+byz^{2}=0\\ x+z^{2}-wz=0\\ 1+x=0\\ yz+cw+dv=0\\ xy+ev=0 \end{array}\right.$$

when a=−1, b=−3, c=−3, d=1.8, e=−5, the three equilibrium points of system (2) obtained by Equation (4) are $S_{0}=[0,0,0,0,0]$, $S_{1}=[\frac{4\sqrt{66}}{3},\frac{4\sqrt{66}}{3},32,\frac{128\sqrt{66}}{9},\frac{128\sqrt{66}}{81}]$, $S_{2}=[-\frac{4\sqrt{66}}{3},-\frac{4\sqrt{66}}{3},32,-\frac{128\sqrt{66}}{9},-\frac{128\sqrt{66}}{81}]$. The balance point is $S_{0}$ and the corresponding eigenvalues are $\lambda_{1}=0,\lambda_{2}=0,\lambda_{3}=-1,\lambda_{4}=-3,\lambda_{5}=-5$. Therefore, $S_{0}$ is a stable focus, the balance point is $S_{1}$, and the corresponding eigenvalues are $\lambda_{1}=25.7790,\lambda_{2}=1.9099,\lambda_{3}=$ $-12.5696,\lambda_{4,5}=-12.0596\pm 81.956i$, where $\lambda_{1},\lambda_{2}$ are positive numbers. Therefore, the balance point $S_{1}$ is an unstable saddle focus, the balance point is $S_{2}$, and the corresponding eigenvalues are $\lambda_{1}=-0.8878,\lambda_{2}=-8.7718,\lambda_{3}=-73.2231,\lambda_{4,5}=36.9413\pm 77.9941i,$ where the real part of $\lambda_{4,5}$ is positive, and the balance point $S_{2}$ is an unstable saddle focus.

### 2.3. Time Series Chart

**Figure 1:**
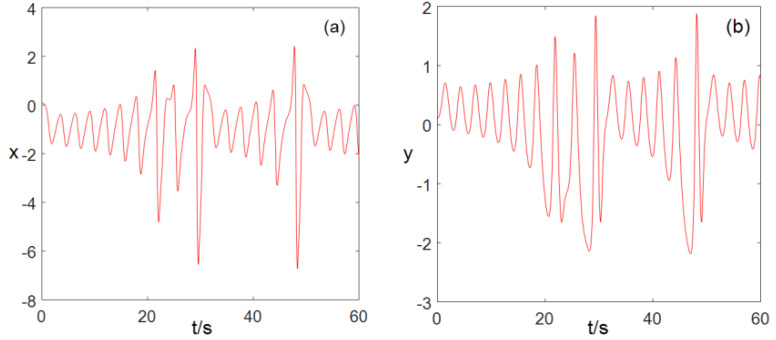

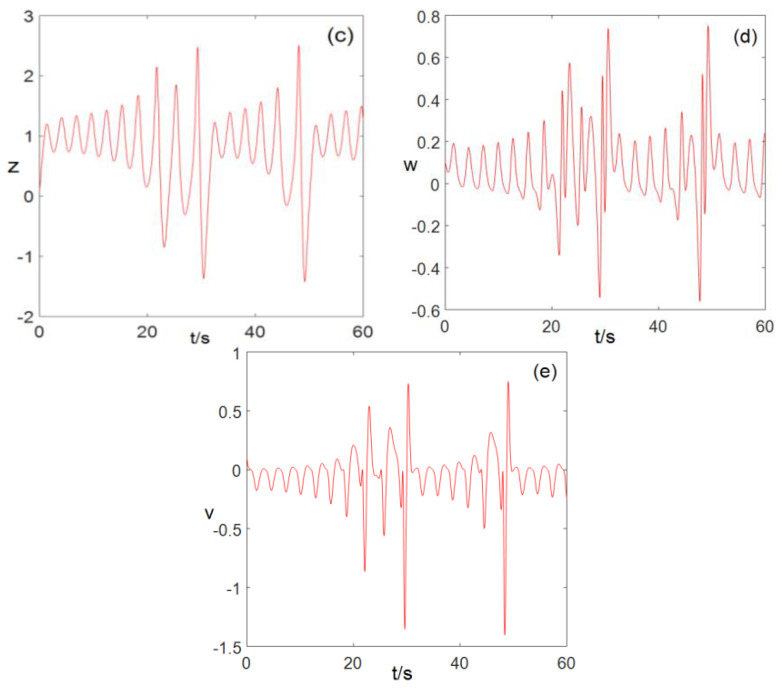
Time series diagram (**a**) x−t time series, (**b**) y−t time series, (**c**) z−t time series, (**d**) w−t time series, (**e**) v−t time series.

When a=−1, b=−3, c=−3, d=1.8, e=−5, the sequence diagram of the values x,y,z,w,v changes over time t, as shown in Figure 1. We can see that when the system parameters a=−1, b=−3, c=−3, d=1.8, e=−5, system (2) is in a chaotic state.

### 2.4. Phase Diagram Analysis

**Figure 2:**
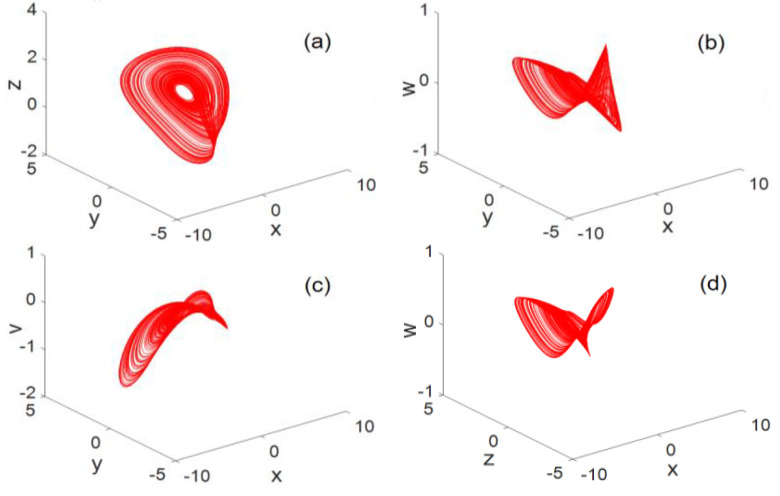

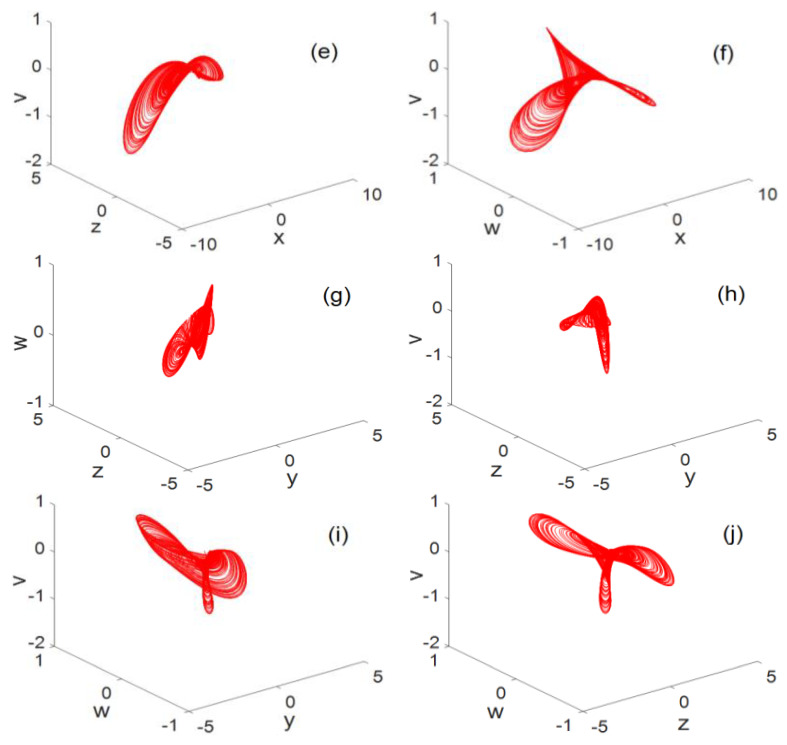
3D phase diagram (**a**) x−y−z 3D map, (**b**) x−y−w 3D map, (**c**) x−y−v 3D map, (**d**) x−z−w 3D map, (**e**) x−z−v 3D map, (**f**) x−w−v 3D map, (**g**) y−z−w 3D map, (**h**) y−z−v 3D map, (**i**) y−w−v 3D map, (**j**) z−w−v 3D map.

**Figure 3:**
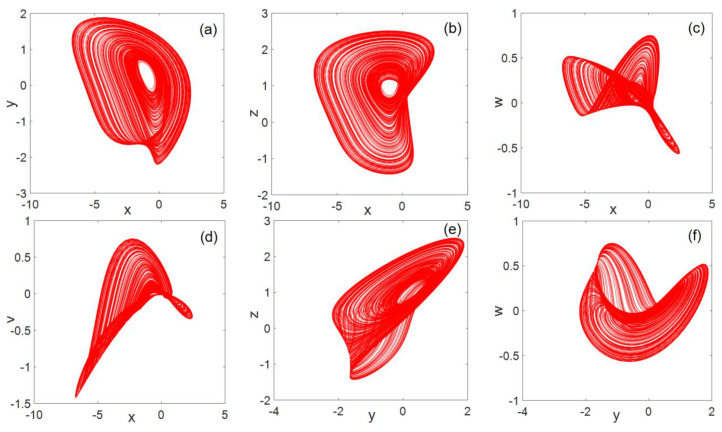

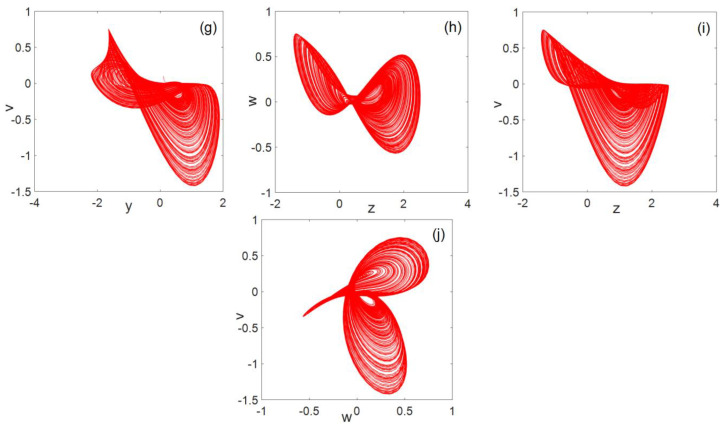
Plane phase diagram (**a**) x−y flat, (**b**) x−z flat, (**c**) x−w flat, (**d**) x−v flat, (**e**) y−z flat, (**f**) y−w flat, (**g**) y−v flat, (**h**) z−w flat, (**i**) z−v flat, and (**j**) w−v flat.

For system parameters a=−1, b=−3, c=−3, d=1.8, e=−5, the 3D phase diagram generated by system (2) is shown in Figure 2. The resulting planar phase diagram is shown in Figure 3. It can be clearly seen from Figure 2 and Figure 3 that the chaotic system can generate three-leaf chaotic attractors in multiple directions.

### 2.5. Bifurcation Diagram

**Figure 4:**
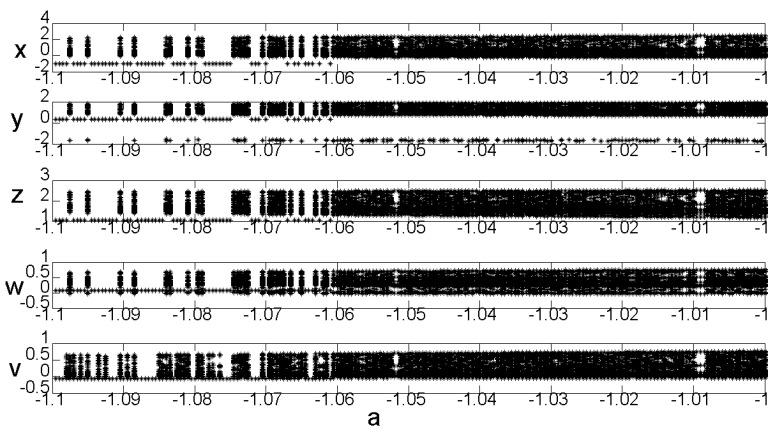
System (2) bifurcation diagram with variable a.

For the equation parameters b=−3, c=−3, d=1.8, e=−5, the bifurcation diagram of the change in parameter a is shown in Figure 4. Figure 4 shows that, when the parameter a∈(−1.1,−1), system (2) is in a chaotic state.

**Figure 5:**
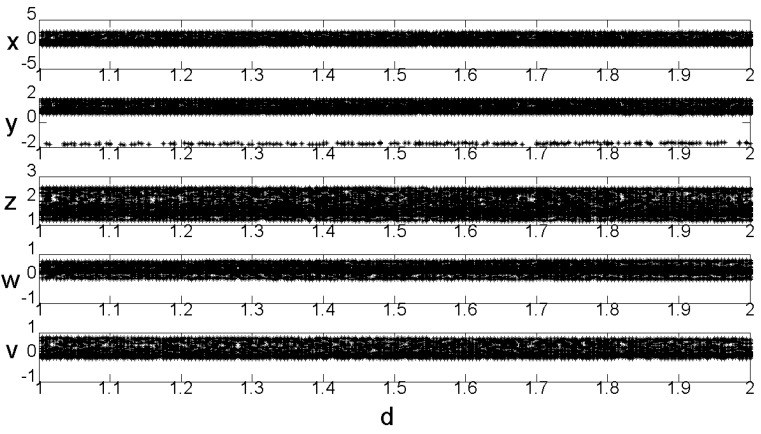
System (2) bifurcation diagram with variable d.

For equation parameters a=−1, b=−3, c=−3, e=−5, the bifurcation diagram of the change in parameter d is shown in Figure 5. As can be seen from Figure 5, when the parameter d∈ (1,2), system (2) is in a chaotic state.

### 2.6. Power Spectrum Analysis

**Figure 6:**
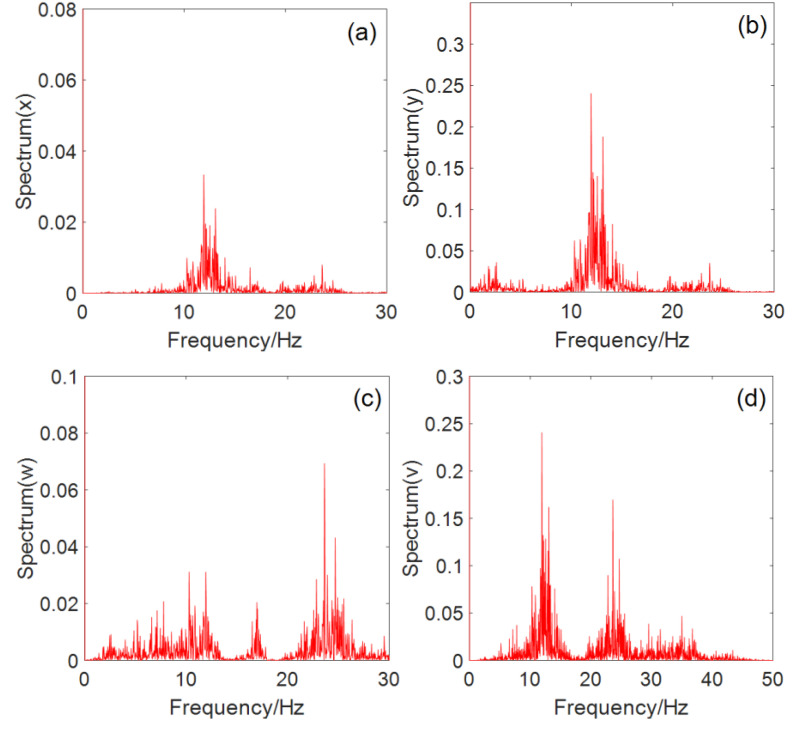
System power spectrum: (**a**) power spectrum of the x sequence, (**b**) power spectrum of the y sequence, (**c**) power spectrum of the w sequence, and (**d**) power spectrum of the v sequence.

The power spectrum of the chaotic sequence is a continuum, and the calculation results of the sequence x,y,w, and v power spectrums of system (2) are shown in Figure 6a–d, respectively. It can be seen from Figure 6 that system (2) is in a chaotic state.

## 3. Circuit Design and Experimental Results

**Figure 7:**
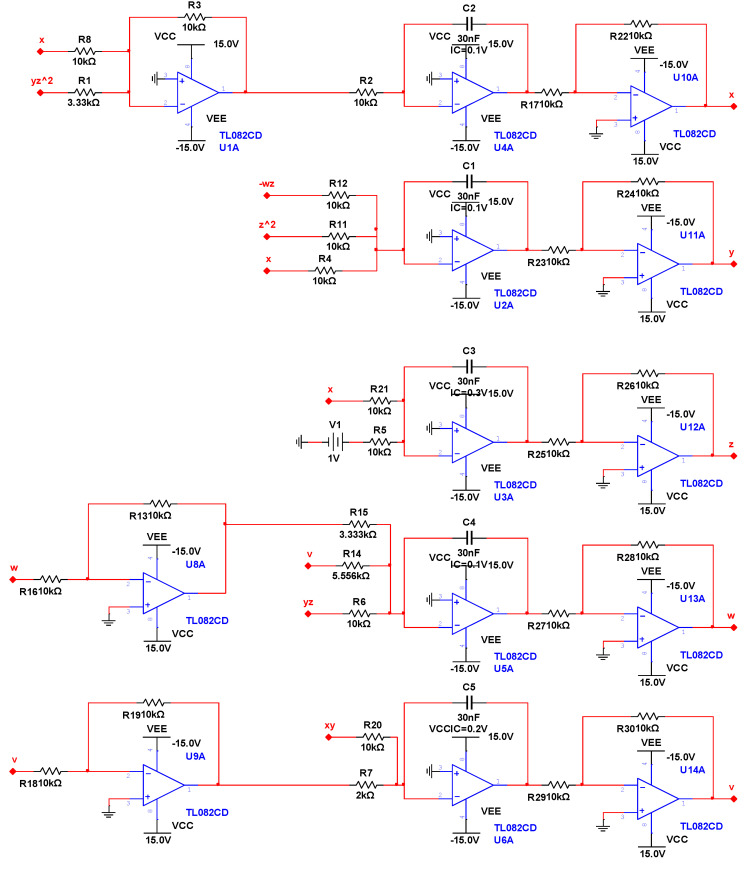
Five-dimensional chaotic system circuit diagrams.

**Figure 8:**
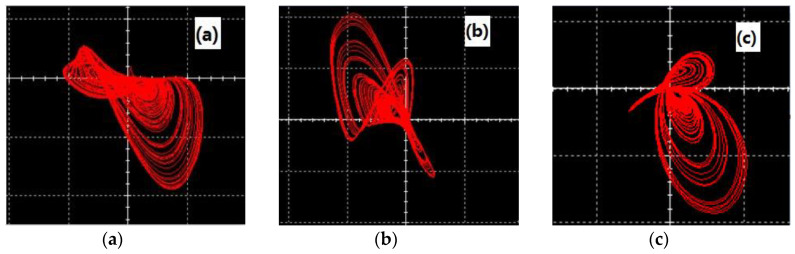
Experimental results for the circuit (**a**) x−w plane, (**b**) w−v plane, and (**c**) w−v plane.

In this section, we design an analog circuit, as shown in Figure 7, which is mainly composed of an inverting adder, integrator, and inverter composed of an operational amplifier TL082CD. The power supply voltage of the operational amplifier TL082CD is E=±15V. This circuit has a simple structure and is easy to implement. The experimental results for this circuit are shown in Figure 8. Figure 8a shows the y−v plane, Figure 8b shows the x−w plane, and Figure 8c shows the w−v plane. It can be seen from the oscilloscope that the experimental results for the circuit for the three-leaf chaotic attractor in each plane were consistent with the results of the numerical simulation experiments.

## 4. Related Information

### 4.1. Convolution Operation

Let

$$h=\left[\begin{array}{cccc} h_{11} & h_{12} & \ldots & h_{1m}\\ h_{21} & h_{22} & \ldots & h_{2m}\\ \vdots & \vdots & \vdots & \vdots \\ h_{m1} & h_{m2} & \ldots & h_{mm} \end{array}\right]$$

is the convolution kernel of m×m,

$$A=\left[\begin{array}{cccc} a_{11} & a_{12} & \ldots & a_{1n}\\ a_{21} & a_{22} & \ldots & a_{2n}\\ \vdots & \vdots & \vdots & \vdots \\ a_{n1} & a_{n2} & \ldots & a_{nn} \end{array}\right]$$

is an n×n matrix, where m<n. Then a n×n matrix C is obtained by a convolution operation between matrix A and convolution kernel h.

$$C=\left[\begin{array}{cccc} c_{11} & c_{12} & \ldots & c_{1n}\\ c_{21} & c_{22} & \ldots & c_{2n}\\ \vdots & \vdots & \vdots & \vdots \\ c_{n1} & c_{n2} & \ldots & c_{nn} \end{array}\right]$$

The convolution operation steps are as follows.

**Step 1:** Extend matrix A to an (n+2)×(n+2) matrix with 0.

$$B=\left[\begin{array}{cccccc} 0 & 0 & 0 & 0 & 0 & 0\\ 0 & a_{11} & a_{12} & \ldots & a_{1n} & 0\\ 0 & a_{21} & a_{22} & \ldots & a_{2n} & 0\\ 0 & \vdots & \vdots & \vdots & \vdots & 0\\ 0 & a_{n1} & a_{n2} & \ldots & a_{nn} & 0\\ 0 & 0 & 0 & 0 & 0 & 0 \end{array}\right]$$

where B(1,1:n+2)=B(1:n+2,1)=B(n+2,1:n+2)=B(1:n+2,n+2)=0, B(2:n+1,2: n+1)=A.

**Step2:** Obtain matrix $C={\left(c_{ij}\right)}_{nn}$ using a convolution operation between matrix A and convolution kernel h, where

(5) $$c_{ij}=\sum_{p,q=1}^{p,q=m}h_{pq}\times B(p+(i-1),q+(j-1)),\;i=1,2,\cdots ,n,\;j=1,2,\cdots n,$$

### 4.2. “Same OR” Operation

The “same OR” operation and the “exclusive OR” operation have the same effect. The “same OR” operation is defined as follows: when the input variables are the same, the output is 1, and when the input variables are different, the output is 0. The calculation results are presented in Table 1.

## 5. Algorithm Descriptions 

### 5.1. Encryption Algorithm Description

**Figure 9:**
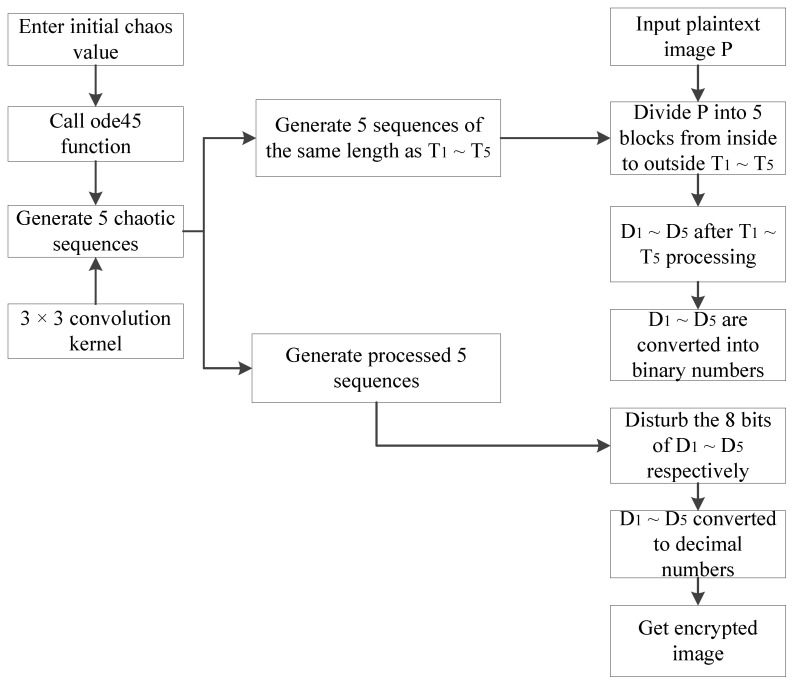
Encryption flow chart.

The flow chart of the encryption is shown in Figure 9.

Given an M×N grayscale image A, the encryption steps are as follows.

**Step 1:** Input a grayscale image A, initial value of the chaotic system $y_{0}=[0.6,0.1,0.2,0.5,0.4]$, and the step size L=0.01. 

**Step 2:** Find the total iteration time T=(250+P×P)×L, where P=max(M,N).

**Step 3:** Call the ode45 function, iterate system (2), and generate five chaotic sequences.

**Step 4:** The five chaotic sequences are treated separately as follows.

(6) $$A1(:,:,i)=(reshape(y(250:249+M\times N,i),M,N))\times 10^{11},i=1,2,3,4,5$$

where y(250:(249+M×N),i) is the M×N value that starts from the 250th value of the chaotic sequence y(:,i). Use $(reshape(y(250:\left(249+M\times N\right),i),M,N))\times 10^{11}$ to convert the fetched M×N values into an M×N matrix, and then multiply each element value of the matrix by $10^{11}$

**Step 5:** Given a convolution kernel of 3×3.

**Step 6:** For the five M×N matrices A1(:,:,i), i=1,2,3,4,5 obtained in step 4, apply the convolution operation with the given convolution kernel to obtain five M×N matrices Y(:,:,i), i=1,2,3,4,5.

**Step 7:** Divide grayscale image A into five regions starting from the center area in the proportion l=M:N. Input the value of $m_{1}$ using $n_{1}=m_{1}÷l$, and obtain *n*_1_. The formulas for *m_i_* and *n_i_* are as follows.

(7) $$\left\{ \begin{array}{l} m_{i+1}=m_{i}+\frac{1}{4}\left(round\left(\frac{M}{2}\right)-m_{1}\right)\\ n_{i+1}=n_{i}+\frac{1}{4}\left(round\left(\frac{N}{2}\right)-n_{1}\right) \end{array}\right.,\;i=1,2,3$$

**Figure 10:**
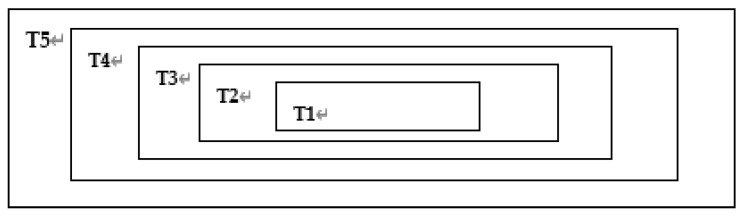
Division of grayscale image A.

There are *T*1 = *m*_1_ × *n*_1_, *T*2 = *m*_2_ × *n*_2_, *T*3 = *m*_3_ × *n*_3_, *T*4 = *m*_4_ × *n*_4_, *T*5 = *M* × *N* as shown in Figure 10.

**Figure 11:**
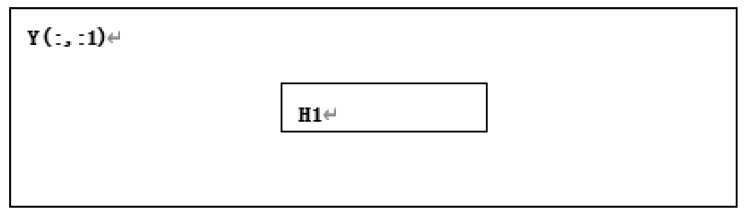
H1 divided from Y(:,:,1).

**Step 8:** Divide the region H1 in matrix Y(:,:,1) according to the starting point and size of T1 in grayscale image A, as shown in Figure 11.

**Step 9:** Convert matrix T1 and matrix H1 sums of one row and $\left(m_{1}\times n_{1}\right)$ column matrix T11 and H11, respectively. H11 is treated as follows.

(8) $$HH=mod(round(H11\times 10^{-7}),256)$$

**Step 10:** Combine T11 and HH, and perform a bitwise “XOR” operation to obtain matrix B1.

**Step 11:** Process H11 as follows to obtain M1 and M2.

(9) $$\left\{ \begin{array}{l} M1=mod(round(H11\times 10^{4}),7)+1\\ M2=mod(round(H11(end-7:end)\times 10^{4}),m_{1}\times n_{1}-1)+1 \end{array}\right.$$

**Step 12:** Convert matrix B1 into binary matrix F1.

**Step 13:** Disturb each line of binary numbers in each row of F1, and then obtain matrix C1. 

The disturbance formula is as follows.

(10) $$C1(i1,:)=circshift(F1(i1,:),M1(i1),2),\;i1=1,2,3,\ldots ,m_{1}\times n_{1}$$

where F1(i1,:) denotes all the columns of row i1 of matrix A and circshift(A,k,2) moves all elements of row vector A clockwise by k units.

**Step 14:** Disturb all column elements of C1, and then obtain C2. The disturbance formula is as follows.

(11) C2(:,i2)=circshift(C1(:,i2),M2(i2),1), i2=1,2,3,4,5,6,7,8

where C1(:,i2) denotes all the rows of column i2 of matrix *C*1 and *circshift*(*A*,*k*,1) moves all the elements of column vector *A* clockwise by *k* units.

**Step 15:** Convert binary number matrix *C*2 to decimal number matrix *D*1.

**Figure 12:**
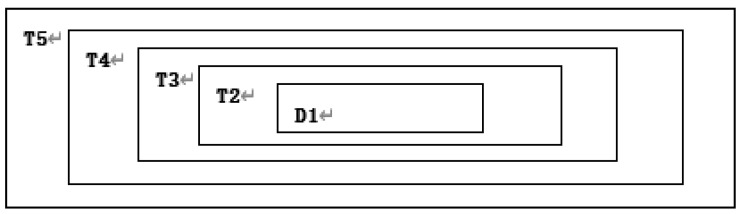
Subtraction after replacing T1 with D1.

**Step 16:***D*1 is used to replace the area *T*1. The results are shown in Figure 12.

**Figure 13:**
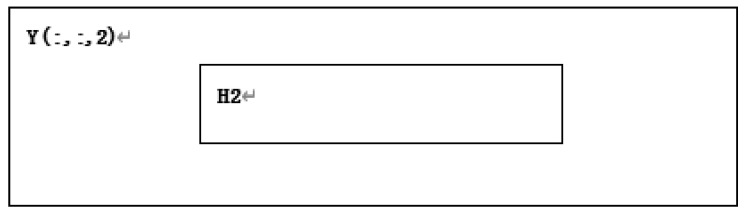
H2 divided in Y(:,:,2).

**Step 17:** The area H2 is taken out according to T2 in the starting point and size in A from Y(:,:,2), as shown in Figure 13.

**Step 18:** Convert matrix T2 and matrix H2 sums of one row and $\left(m_{2}\times n_{2}\right)$ column matrix T22 and H22, respectively. H22 is treated as follows.

(12) $$HH1=mod(round(H22\times 10^{-7}),256)$$

**Step 19:** Combine T22 and HH1, and perform a bitwise “same OR” operation to obtain matrix B2

**Step 20:** Process H22 to obtain M3 and M4 as follows.

(13) $$\left\{ \begin{array}{l} M3=mod(round(H22\times 10^{4}),7)+1\\ M4=mod(round(H22(end-7:end)\times 10^{4}),m_{2}\times n_{2}-1)+1 \end{array}\right.$$

**Step 21:** Convert matrix B2 into binary matrix F2.

**Step 22:** Scramble each row of binary numbers in F2 to obtain matrix C3. The scrambling formula is as follows.

(14) $$C3(i3,:)=circshift(F2(i3,:),M3(i3),2),\;i3=1,2,3,\ldots ,m_{2}\times n_{2}$$

**Step 23:** Disturb all the column elements of C3 to obtain C4. The scrambling formula is as follows.

(15) C4(:,i4)=circshift(C3(:,i4),M4(i4),1), i4=1,2,3,4,5,6,7,8

**Step 24:** Convert binary number matrix C4 to decimal number matrix D2.

**Figure 14:**
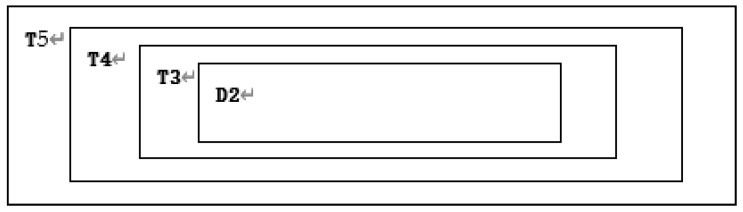
Subtraction after replacing T2 with D2.

**Step 25:** Replace area T2 with D2. The result is shown in Figure 14.

**Figure 15:**
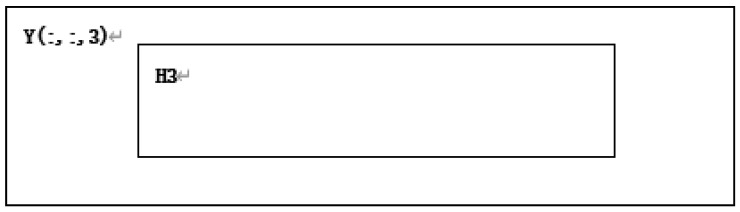
Y(:,:,3) is divided to obtain H3.

**Step26:** According to T3 from the starting point and size in the A, take the area H3 from Y(:,:,3), as shown in Figure 15.

**Step 27:** Convert the matrices T3 and H3 into matrices T33 and H33 with one row and $\left(m_{3}\times n_{3}\right)$ columns, respectively, and process H33 as follows.

(16) $$HH2=mod(round(H33\times 10^{-7}),256)$$

**Step 28:** Combine T33 and HH2, and perform a bitwise “same OR” operation to obtain matrix B3.

**Step 29:** Process H33 as follows to obtain M5 and M6.

(17) $$\left\{ \begin{array}{l} M5=mod(round(H33\times 10^{4}),7)+1\\ M6=mod(round(H33(end-7:end)\times 10^{4}),m_{3}\times n_{3}-1)+1 \end{array}\right.$$

**Step 30:** Convert matrix B3 into binary matrix F3.

**Step 31:** Scramble the binary numbers in each row of F3 to obtain matrix C5. The scrambling formula is as follows.

(18) $$C5(i5,:)=circshift(F3(i5,:),M5(i5),2),\;i5=1,2,3,\ldots ,m_{3}\times n_{3}$$

**Step 32:** Disturb all the column elements of C5 to obtain C6. The scrambling formula is as follows.

(19) C6(:,i6)=circshift(C5(:,i6),M6(i6),1), i6=1,2,3,4,5,6,7,8

**Step 33:** Convert binary number matrix C6 to decimal number matrix D3.

**Figure 16:**
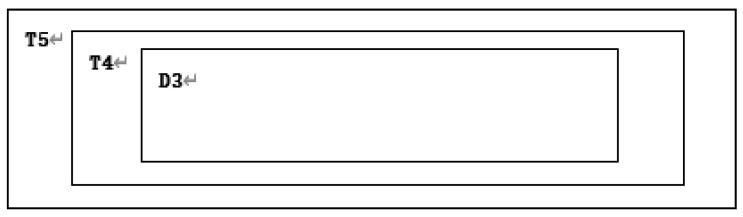
Subtraction after replacing T3 with D3.

**Step 34:** Replace area T3 with D3. The result is shown in Figure 16.

**Figure 17:**
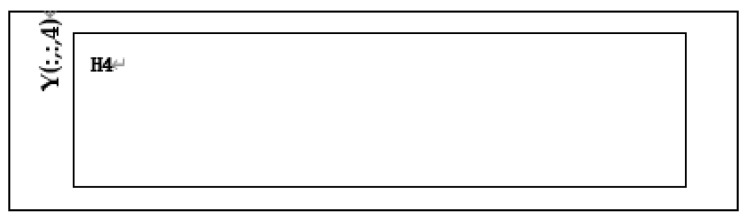
Y(:,:,4) is divided to obtain H4.

**Step 35:** Take region H4 from Y(:,:,4) according to the starting point and size of T4 in A, as shown in Figure 17.

**Step 36:** Convert matrices T4 and H4 into matrices T44 and H44 with one row and $\left(m_{4}\times n_{4}\right)$ columns, respectively, and process H44 as follows.

(20) $$HH3=mod(round(H44\times 10^{-7}),256)$$

**Step 37:** Combine T44 and HH3, and perform a bitwise “XOR” operation to obtain matrix B4.

**Step 38:** Process H44 to obtain M7 and M8 as follows.

(21) $$\left\{ \begin{array}{l} M7=mod(round(H44\times 10^{4}),7)+1\\ M8=mod(round(H44(end-7:end)\times 10^{4}),m_{4}\times n_{4}-1)+1 \end{array}\right.$$

**Step 39:** Convert matrix B4 into binary matrix F4.

**Step 40:** Scramble the binary numbers in each row in F4 to obtain matrix C7. The scrambling formula is as follows.

(22) $$C7(i7,:)=circshift(F4(i7,:),M7(i7),2),\;i7=1,2,3,\ldots ,m_{4}\times n_{4}$$

**Step 41:** Disturb all the column elements of C7 to obtain C8. The scrambling formula is as follows.

(23) C8(:,i8)=circshift(C7(:,i8),M8(i8),1), i8=1,2,3,4,5,6,7,8

**Step42:** Convert binary number matrix C8 to decimal number matrix D4.

**Figure 18:**
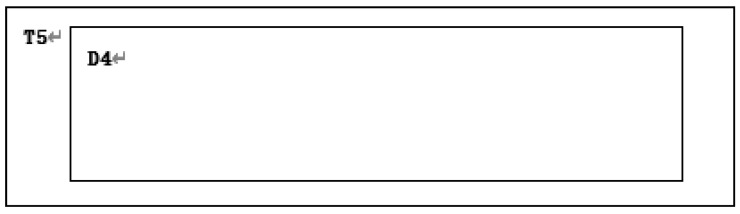
Subtraction after replacing T4 with D4.

**Step43:** Replace area T4 with D4. The result is shown in Figure 18.

**Step 44:** Convert matrix T5 and Y(:,:,5) to matrix T55 and H55 with one row and (M×N) columns, respectively, and H55 is treated as follows.

(24) $$HH4=mod(round(H55\times 10^{-7}),256)$$

**Step 45:** Combine T55 and HH4, and perform a bitwise “same OR” operation to obtain matrix B5.

**Step 46:** Process H55 as follows to obtain M9 and M10.

(25) $$\left\{ \begin{array}{l} M9=mod(round(H55\times 10^{4}),7)+1\\ M10=mod(round(H55(end-7:end)\times 10^{4}),M\times N-1)+1 \end{array}\right.$$

**Step 47:** Convert matrix B5 into binary matrix F5.

**Step 48:** Scramble the binary numbers in each row of F5 to obtain matrix C9. The scrambling formula is as follows.

(26) C9(i9,:)=circshift(F5(i9,:),M9(i9),2), i9=1,2,3,…,M×N

**Step 49:** Disturb all the column elements of C9. The scrambling formula is as follows.

(27) C10(:,i10)=circshift(C9(:,i10),M10(i10),1), i10=1,2,3,4,5,6,7,8

**Figure 19:**
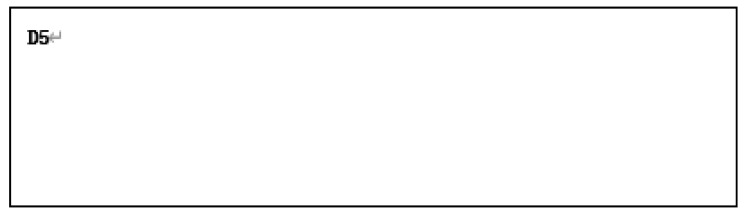
Encrypted image.

**Step 50:** Convert binary number matrix C10 to decimal number matrix D5, and D5 is the final encrypted image. The result is shown in Figure 19.

### 5.2. Decryption Algorithm Description

**Step 1:** Input the initial value of the chaotic system $y_{0}=[0.6,0.1,0.2,0.5,0.4]$ and step size L= 0.02, and find the total iteration time T=(250+P×P)×L, where P=max(M,N).

**Step 2:** Call the ode45 function, iterate system (2), and generate five chaotic sequences.

**Step 3:** The five chaotic sequences are treated as follows.

(28) $$A1(:,:,i)=(reshape(y(250:249+M\times N,i),M,N))\times 10^{11},\;i=1,2,3,4,5$$

where y(250:(249+M×N),i) is the M×N value starting from the 250th value of chaotic sequence y(:,i). Use $(reshape(y(250:\left(249+M\times N\right),i),M,N))\times 10^{11}$ to convert the fetched M×N values into an M×N matrix, and then multiply each element value of the matrix by $10^{11}$.

**Step 4:** Regarding the matrix A1(:,:,i), i=1,2,3,4,5 of M×N, the five obtained in the previous step convolution operation with the given convolution kernel of the encryption algorithm include five matrix Y1(:,:,i), i=1,2,3,4,5 of M×N are obtained.

**Figure 20:**
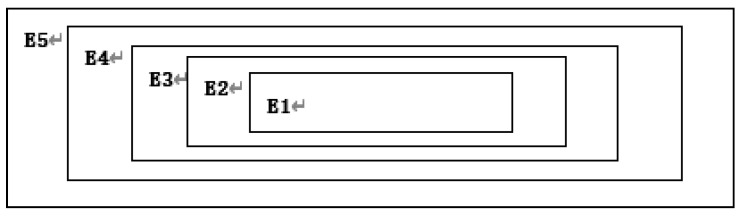
Division diagram for D5.

**Step 5:** Divide the encrypted image D5 of M×N into five regions in proportion to the middle region, with l=M:N, $E1=(m_{1}\times n_{2}),E2(m_{2}\times n_{2}),E3(m_{3}\times n_{3}),E4(m_{4}\times n_{4}),E5(M\times N)$, as shown in Figure 20.

**Step6:** Restore E5,E4,E3,E2,E1 in turn to obtain decrypted image A.

## 6. Experimental Results and Analysis

### 6.1. Experiment Platform

The PC configuration was as follows: Intel(R) Core (TM) i5-6500 CPU @ 3.70 GHz 3.70 GHz, memory 8 GB, and Windows 7 64-bit operating system. The above encryption algorithm was implemented in a program in MATLAB R2014a.

### 6.2. Experimental Result

**Figure 21:**
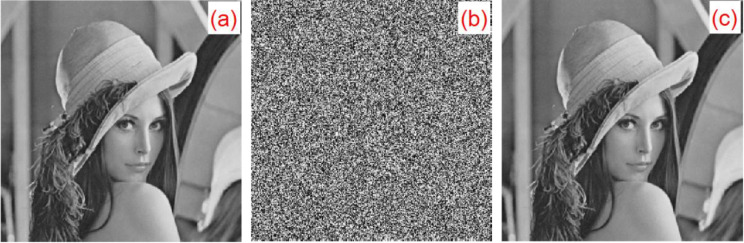

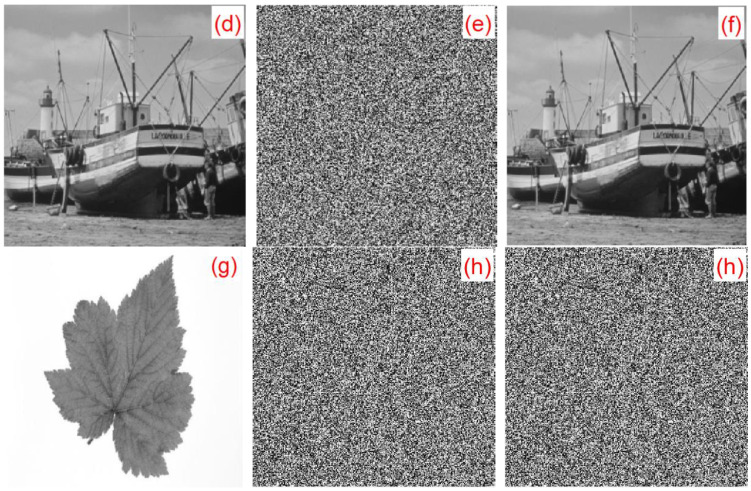
Encrypted/decrypted experimental results: (**a**) Lena original image, (**b**) Lena encrypted image, (**c**) Lena decrypted image, (**d**) boat original image, (**e**) boat encrypted image, (**f**) boat decrypted image, (**g**) leaf original image, (**h**) leaf encrypted image, and (**i**) leaf decrypted image.

For the experiment, six types of grayscale images of classic images were selected: Lena, boat, baboon, peppers, couple, and leaf, which were all 256×256. This algorithm is also applicable to grayscale images of any sizes. The plaintext image, encrypted image, and decrypted image are shown in Figure 21.

### 6.3. Key Space Analysis

The size of the key space is one of the most important factors that determines the strength of the image encryption algorithm. The larger the key space, the stronger the ability to resist brute force 

attacks. The secret keys of this proposed encryption algorithm include five initial values $y_{0}=[0.6,0.1,0.2，0.5,0.4]$ and five system parameters a,b,c,d,e. Since the precision is $10^{-15}$ by computer with accuracy, the key space is ${\left(10^{15}\right)}^{10}=10^{150}$. In addition, when the convolution kernel size is 3×3, the secret key also needs to consider nine convolution kernel parameters. Therefore, the total key space of the algorithm is much larger than $10^{150}>2^{100}$. For a security encryption algorithm, its key space should be larger than $2^{100}$ [21]. Therefore, this algorithm was sufficiently secure.

### 6.4. Convolution Nuclear Sensitivity Analysis

**Figure 22:**
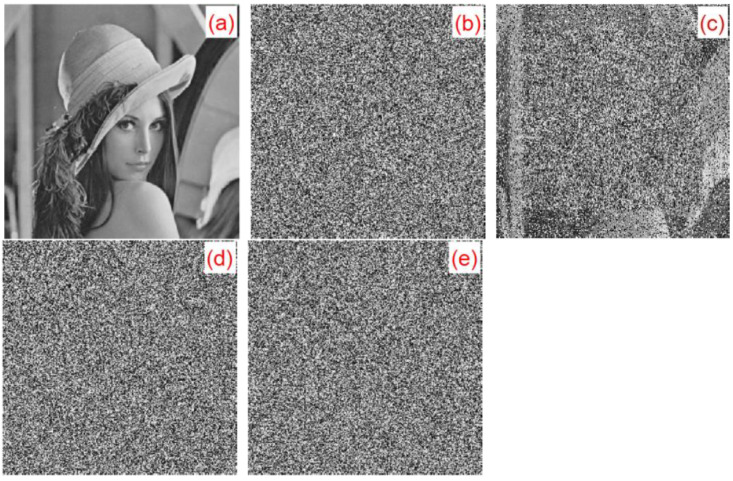
$c_{1},c_{2},c_{3}$ decryption map: (**a**) Lena plaintext image, (**b**) c corresponding decrypted image, (**c**) $c_{1}$ corresponding decrypted image, (**d**) $c_{2}$ corresponding decrypted image, and (**e**) $c_{3}$ corresponding decrypted image.

In the encryption process, we multiplied each element of the five chaotic sequences produced by 10^11^ and performed convolution operations with the convolution kernel of 3×3. The convolution kernel of 3×3 in this algorithm was c=[1,2,3;4,5,6;7,8,9]. In the decryption process, when any of the parameters in the convolution kernel were slightly changed, the original image could not be successfully decrypted. When any parameter in the convolution kernel changed slightly with $10^{-15}$, the decrypted image was blurred, but the outline could be seen, as shown in Figure 22c. When any of the parameters in the convolution kernel was slightly changed with $10^{-14}$, the decrypted image could not substantially display the plaintext image information, as shown in Figure 22d. When any parameter in the convolution kernel changed slightly with $10^{-13}$, the plaintext image information could not be solved at all, as shown in Figure 22e. Taking the Lena image as an example, we made a slight change to the parameters of the second row and the second column of the convolution kernel: $c_{1}=[1,2,3;4,5.000000000000001,6;7,8,9]$, $c_{2}=[1,2,3;4,5.00000000000001,6;7,8,9]$, $c_{3}=[1,2,3;4,$ 5.0000000000001,6;7,8,9]. The plaintext images, ciphertext images, and the corresponding decrypted images of $c_{1}$, $c_{2}$, and $c_{3}$ are shown in Figure 22.

**Figure 23:**
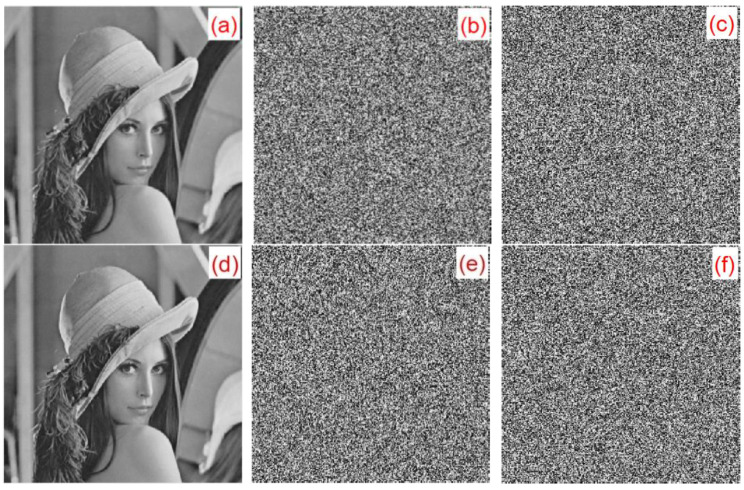
Convolution nuclear sensitivity experiment analysis chart: (**a**) Plaintext image, (**b**) Ciphertext $C_{0}$ (convolution kernel $c_{0}$), (**c**) Ciphertext $C_{1}$ (convolution kernel $c_{1}$), (**d**) $C_{0}$ correct decryption result, (**e**) $C_{0}$ error decryption result using $c_{1}$, and (**f**) $C_{1}$ error decryption result using $c_{0}$.

A small change in the convolution kernel can lead to a great change in the ciphertext. In this study, only the Lena image was used as an example. Figure 23 shows the sensitivity of this algorithm to convolution kernels. Figure 23a is a plaintext image. Figure 23b,c are convolution kernels, $c_{0}=[1,2,3;4,5,6;7,8,9]$ and $c_{1}=[1,2,3;4,5.00000000000001,6;7,8,9]$ for encrypted ciphertext image $C_{0}$ and $C_{1}$, respectively. Figure 23d is the result of the correct decryption for $C_{0}$ using $c_{0}$. Figure 23e,f show the results of $C_{0}$ and $C_{1}$ using the wrong convolution kernel $c_{1}$ and $c_{0}$ decryption, respectively. Figure 23 illustrates that, despite only minor changes between the convolution kernels $c_{0}$ and $c_{1}$, the ciphertext images $C_{0}$ and $C_{1}$ could not be properly decrypted with the convolution kernels.

The difference between the two images can also be measured by the pixel change rate (NPCR) and the normalized mean change intensity (UACI), which are described as Equations (29) and (30).

(29) $$NPCR=\frac{\sum_{i,j}^{M,N}D(i,j)}{M\times N}\times 100\%$$

(30) $$UACI=\frac{\sum_{i,j}^{M,N}\left|C_{1}(i,j)-C_{2}(i,j)\right|}{M\times N\times 255}\times 100\%$$

where $D(i,j)=\left\{ \begin{array}{l} 1,C_{1}(i,j)\neq C_{2}(i,j)\\ 0,C_{1}(i,j)=C_{2}(i,j) \end{array}\right.$; M,N is the size of the image. $C_{1}(i,j)$ and $C_{2}(i,j)$ are the pixel values at position (i,j). The larger the values of NPCR and UACI, the greater the difference between the two images. To better evaluate the sensitivity of the convolution kernel, we use Equations (29) and (30) to calculate the NPCR and UACI of the encrypted images obtained by the slightly changed convolution kernels $c_{1},c_{2},c_{3},c_{4},c_{5}$ and the encrypted image obtained by the original convolution kernel $c_{0}$. There are $c_{1}=[1,2,3;4,5.000000000000001,6;7,8,9]$, $c_{2}=[1,2,$ 3;4,5.00000000000001,6;7,8,9], $c_{3}=[1,2,3;4,5.0000000000001,6;7,8,9]$, $c_{4}=[1,2,3;4,$ 5.00000000001,6;7,8,9], $c_{5}=[1,2,3;4,5.00000000001,6;7,8,9]$, $c_{0}=[1,2,3;4,5,6;7,8,9]$. The NPCR and UACI values between the obtained encrypted images are shown in Table 2. Table 2 shows that using the convolution kernel operation can greatly increase the key space of the algorithm.

### 6.5. Key Sensitivity Analysis

**Figure 24:**
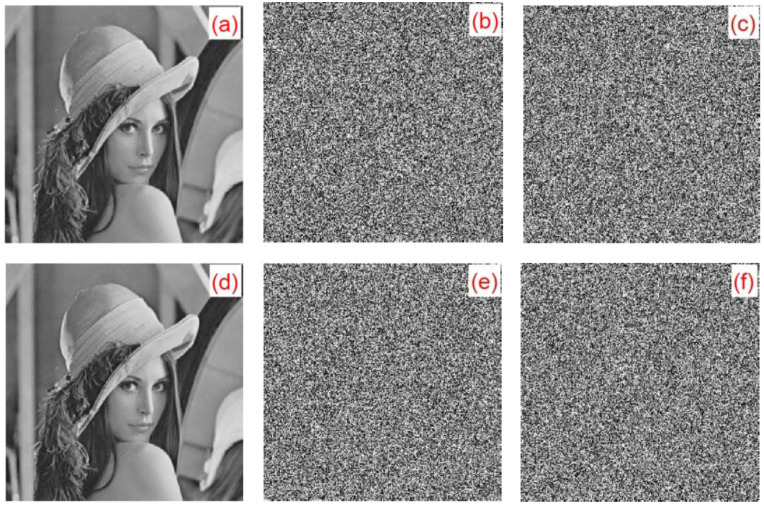
Key sensitivity experiment analysis chart: (**a**) plaintext image, (**b**) ciphertext $Y_{0}$ (key is $y_{0}$), (**c**) ciphertext $Y_{1}$ (key is $y_{1}$), (**d**) $Y_{0}$ Correct decryption result, (**e**) error decryption result $Y_{0}$ using $y_{1}$, and (**f**) error decryption result $Y_{1}$ using $y_{0}$.

A small change in the key results in a great change in the ciphertext, which is the key sensitivity. In the experiment, the Lena image was considered as an example. Figure 24 shows the sensitivity of the algorithm to the initial key. Figure 24a is a plaintext image. Figure 24b,c show the results of using keys $y_{0}=[0.6,0.1,0.2,0.5,0.4]$ and $y_{1}=[0.600000000000001,0.1,0.2,0.5,0.4]$ for encrypted ciphertext image $Y_{0}$ and $Y_{1}$, respectively. Figure 24d shows the result of the correct decryption of $Y_{0}$ using $y_{0}$. Figure 24e,f show the results of $Y_{0}$ and $Y_{1}$ using the wrong decryption keys $y_{1}$ and $y_{0}$, respectively. Figure 24 illustrates that, despite only minor changes between the keys $y_{0}$ and $y_{1}$, the ciphertext images $Y_{0}$ and $Y_{1}$ could be decrypted correctly using the corresponding keys $y_{1}$ and $y_{0}$.

To better evaluate the key sensitivity of the algorithm, we tested the NPCR and UACI values between the keys $y_{0}=[0.6,0.1,0.2,0.5,0.4]$ and y=[0.600000000000001,0.1,0.2,0.5,0.4] for the encrypted image using Equations (29) and (30). The test values in this study and those in the literature [4,14,17] are shown in Table 3. Table 3 shows that the algorithm had good test results, so the encryption algorithm proposed in this paper has good key sensitivity.

### 6.6. Information Entropy Analysis

Information entropy is an important indicator of randomness, which reflects the distribution of gray values of images. The more uniform the gray value distribution, the larger the information entropy of the image. The information entropy calculation formula of an image is shown below.

(31) $$H(C)=-\sum_{i=1}^{L}p(x_{i})\log_{2}p(x_{i})$$

where $p(x_{i})$ is the probability of C and L is the total number of $x_{i}$. For grayscale images, the theoretical maximum value of information entropy is 8. The closer the image information entropy is to the theoretical maximum, the more random the image pixel gray value distribution is. The information entropy before and after the encryption of Lena, baboon, boat, peppers, couple, and leaf is shown in Table 4. The simulation results show that the pixel value distribution of the encrypted image was very uniform, and the algorithm had a good encryption effect.

Considering the Lena image as an example, the information entropy after encrypting the image was compared with the information entropy in the literature [4,11,12,13,17]. The experimental results are shown in Table 5. Table 5 shows that the algorithm had a better encryption effect.

Global and local entropy [22] values of the encrypted image are tabulated in Table 6. From Table 6, it is clear that both the entropy values are closer to the optimal theoretical values (≈8). Furthermore, the obtained results are compared against the critical values at 5%, 1%, and 0.1% significance. From the above discussion, it can be concluded that the proposed encryption algorithm possesses high randomness and is robust against a statistical attack.

### 6.7. Histogram Analysis

**Figure 25:**
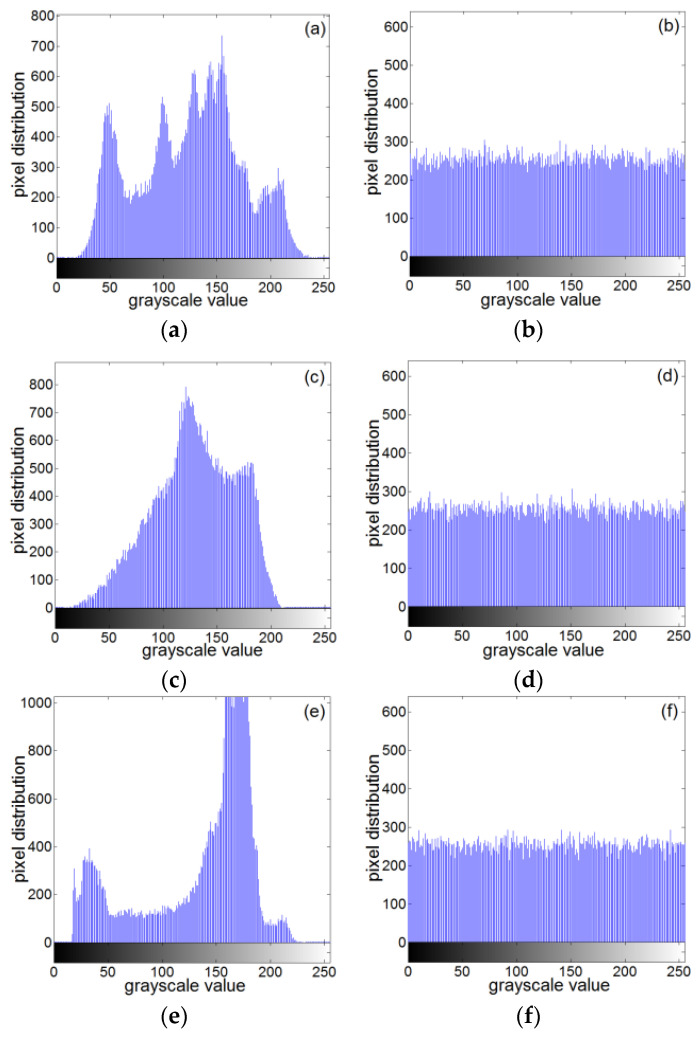
Histogram of the plaintext image and ciphertext image: (**a**) histogram of Lena plaintext, (**b**) histogram of Lena ciphertext, (**c**) histogram of baboon plaintext, (**d**) histogram of baboon ciphertext, (**e**) histogram of the clear text of the boat, and (**f**) histogram of the boat ciphertext.

A histogram can reflect the distribution of image pixel values very well. The flatter the histogram is, the more uniform the pixel value distribution is. Figure 25 shows histograms of the original images for Lena, baboon, and boat, and encrypted images.

### 6.8. Histogram Statistics

The variance and standard deviation are metrics of dispersion implemented to support the results of visual inspection in graphic histograms. They measure how much the elements of a set of data vary with respect to each other around the mean. Two datasets may have the same average value (mean), but the variations can be drastically different [23].

The variance calculates the average difference between each of the values with respect to their central point (mean $\bar{x}$). This average is calculated by squaring each of the differences and calculating its mean again. The squaring process is used to eliminate the negative signs and to increase the variance of dispersed (non-uniform) datasets. On the other hand, the more uniform the graphic histogram is, the lower the histogram variance is, which is determined with the following expression.

(32) $$\alpha =\frac{1}{256}\sum_{i=1}^{256}{\left(x_{i}-\bar{x}\right)}^{2}$$

where:

(33) $$\bar{x}=\frac{M\times N}{256}$$

x is the frequency for each intensity value from 0–255 grayscale value of the histogram, α is the histogram variance, and $\bar{x}$ is the mean of the histogram. The standard deviation allows us to know the arithmetic average of fluctuations of the dataset with respect to the mean. It is determined with the square root of the histogram variance as follows.

(34) $$\beta =\sqrt{\alpha }$$

where β is the standard deviation of the histogram. In Table 7, the histogram variance and its standard deviation are presented for plain and encrypted images. Table 7 shows that the pixel values of the image encrypted by this algorithm are more uniform. This algorithm has a better encryption effect.

### 6.9. Correlation Analysis of Adjacent Pixels

A feature of digital images is the strong correlation of adjacent pixels. To calculate the correlation of adjacent pixels before and after encryption, 5000 sets of adjacent pixels were randomly selected in the horizontal, vertical, and diagonal directions of the plaintext and ciphertext images. The horizontal, vertical, and diagonal correlation coefficients were calculated using Equations (35).

(35) $$\left\{ \begin{array}{l} E(x)=\frac{1}{K}\sum_{i=1}^{K}x_{i}\\ D(x)=\frac{1}{K}\sum_{i=1}^{K}(x_{i}-E(x){)}^{2}\\ Cov(x,y)=\frac{1}{K}\sum_{i=1}^{K}(x_{i}-E(x))(y_{i}-E(y))\\ r_{xy}=\frac{Cov(x,y)}{\sqrt{D(x)}\sqrt{D(y)}} \end{array}\right.$$

**Figure 26:**
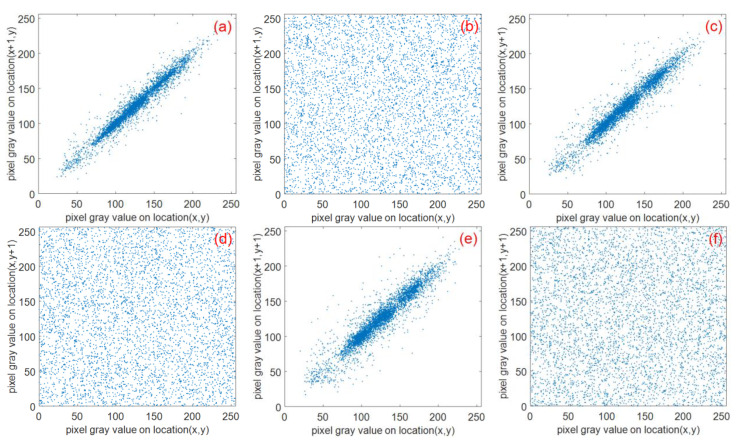
Correlation analysis of the three directions before and after Lena image encryption: (**a**) and (**b**) horizontally adjacent, (**c**) and (**d**) vertically adjacent, (**e**) and (**f**) diagonally adjacent.

The test results are shown in Table 8. The pixel correlation of the Lena plaintext image and ciphertext image in the horizontal direction, vertical direction, and diagonal direction are shown in Figure 26.

### 6.10. Robustness Analysis

#### 6.10.1. Quality Metrics Analysis

Quality evaluation of digital images can use the Mean Squared Error (MSE) and Peak Signal-to-Noise Ratio (PSNR) for measurement [23,24]. The MSE is a parameter to measure the difference between two images, which is described as Equation (36).

(36) $$MSE=\frac{1}{H\times W}\sum_{i=1}^{H}\sum_{j=1}^{W}{\left(X(i,j)-ϒ(i,j)\right)}^{2}$$

where H×W is the size of original image, X(i,j) is the original image, and ϒ(i,j) is the encrypted image. The higher value of MSE represents better encryption quality. This MSE analysis is a useful test for a plain image and encrypted image with pixel values in the range of [0–255]. The PSNR (expressed in logarithmic scale and decibels) determines the ratio between the maximum possible power of a signal and the power of distorting noise that affects the quality of its representation. It is calculated by Equation (37).

(37) $$PSNR=20\log_{10}\left(\frac{255}{\sqrt{MSE}}\right)$$

The smaller the *MSE* value is, the larger the *PSNR* value is, which means that there is a high degree of similarity between the tested images. By calculation, the MSE between the original image and the decrypted image is 0, and the value of PSNR is Inf. In this algorithm, the MSE between the original Lena image and the decrypted image is 77,012, and PSNR is 9.265. The results show that the quality metrics of the tested images is good.

#### 6.10.2. Occlusion Attack Analysis

**Figure 27:**
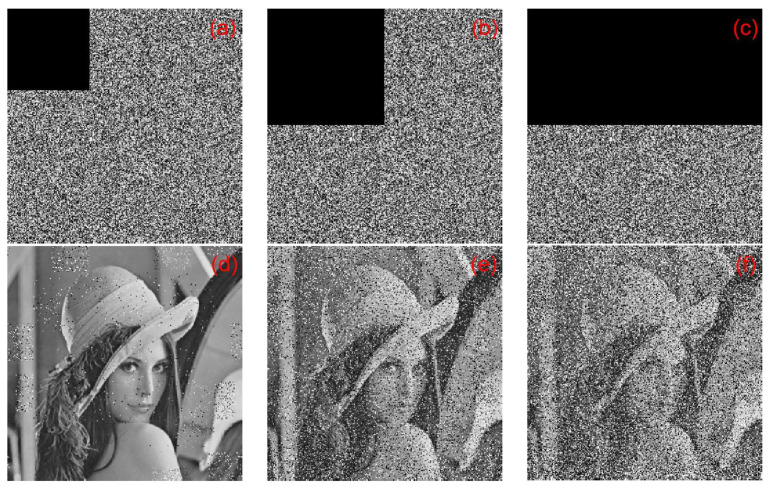
The results of occlusion attack: (**a**) encrypted with 12.5% occlusion, (**b**) encrypted with 25% occlusion, (**c**) encrypted with 50% occlusion, (**d**) decrypted with 12.5% occlusion, (**e**) decrypted with 25% occlusion, and (**f**) decrypted with 50% occlusion.

In an occlusion attack, we choose 12.5%, 25%, and 50% of occlusion in an encrypted image. In Figure 27, the attack results are shown. For 12.5% of occlusion, the MSE value is 7853 and the PSNR value is 9.1802. For 25% of occlusion, the MSE value is 10,148 and the PSNR value is 8.0672. For 50% of occlusion, the MSE value is 13,376 and the PSNR value is 6.8676. The results show that the proposed cryptographic algorithm can effectively resist occlusion attack.

#### 6.10.3. Noise Attack Analysis

**Figure 28:**
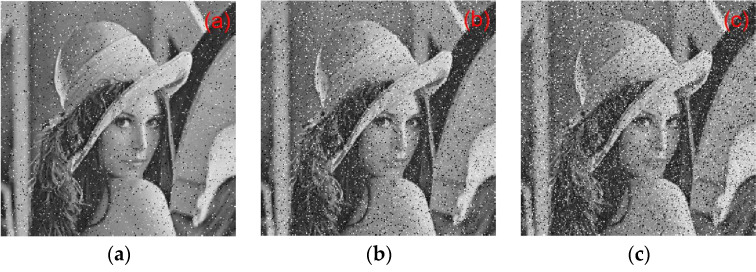
The results of noise attack analysis: (**a**) noise with 10 of intensity, (**b**) noise with 15 of intensity, and (**c**) noise with 20 of intensity.

In order to verify the anti-noise performance of the proposed algorithm, Salt and pepper noise with different intensities was added to the encrypted image. The intensities were 10, 15, and 20, respectively, and they were then decrypted. The results are shown in Figure 28. For 10 of intensity, the MSE value is 8758.9 and the PSNR value is 8.706. For 15 of intensity, the MSE value is 9302.9 and the PSNR value is 8.446. For 20 of intensity, the MSE value is 9866.8 and the PSNR value is 8.189. It can be seen that the original image can be basically recovered after the noise image is decrypted. Therefore, the proposed algorithm has a certain anti-noise attack capability.

## 7. Conclusions

In this paper, a five-dimensional chaotic system was proposed, which had a simple structure and was easy to implement. Basic dynamic analysis of the system was conducted, including the equilibrium point, phase diagram, bifurcation diagram, and power spectrum. Based on the theoretical analysis, a chaotic circuit was designed using the analog device amplifier TL082CD. The consistency of the numerical simulation results confirmed the feasibility of the method. Simultaneously, five chaotic sequences generated by the system were applied to the hybrid image encryption algorithm for physical chaotic encryption and advanced encryption standard encryption. In the algebraic encryption process, we performed convolution operations on five chaotic sequences, which was followed by convolution operations. The latter sequence was applied to the image scaled block encryption, and a numerical simulation experiment was conducted on the hybrid encryption system. The simulation results verified the correctness of the encryption algorithm. Therefore, the encryption algorithm proposed in this paper has a good application prospect in secure communication, particularly digital image encryption.

## Figures and Tables

**Table 1:** Result table for the “same OR” operation.

A(Input)	B(Input)	F(Result)
0	0	1
0	1	0
1	0	0
1	1	1

**Table 2:** Convolution nuclear sensitivity test results.

Lena Image		$\mathrm{Kernel}\;c_{1}$	$\mathrm{Kernel}\;c_{2}$	$\mathrm{Kernel}\;c_{3}$	$\mathrm{Kernel}\;c_{4}$	$\mathrm{Kernel}\;c_{5}$
Index	Pixel Change Rate (NPCR)	0.9716	0.9962	0.9962	0.9963	0.996
	Normalized Mean Change Intensity (UACI)	0.2271	0.3341	0.3335	0.3352	0.3346

**Table 3:** Key sensitivity test results.

Images	Lena		Baboon		Boat		Peppers	
Index	Pixel Change Rate (NPCR)	Normalized Mean Change Intensity (UACI)	NPCR	UACI	NPCR	UACI	NPCR	UACI
Test value	0.9961	0.3356	0.9962	0.3344	0.996	0.3363	0.9962	0.3357
Reference [4]	0.9961	0.3346	-	-	-	-	-	-
Reference [14]	0.9961	0.3346	-	-	-	-	0.9962	0.3341
Reference [17]	0.9952	0.3359	0.991	0.3325	0.9925	0.3339	0.985	0.3295

**Table 4:** Information entropy analysis for plaintext and encrypted images.

Image	Lena	Baboon	Boat	Peppers	Couple
Original Image	7.4832	7.3713	7.1267	7.5715	7.2369
Encrypted Image	7.9978	7.9977	7.997	7.9974	7.9974

**Table 5:** Information entropy comparison.

Image	Original Image Information Entropy	Encrypted Image Information Entropy					
		Algorithm	Reference [4]	Reference [11]	Reference [12]	Reference [13]	Reference [17]
Lena	7.4832	7.9978	7.9967	7.9972	7.9900	7.9959	7.9975

**Table 6:** Global and local entropy analysis.

Image	Global Entropy	Local Entropy No. of Blocks = 20 (Block Size = 44 × 44)	Local Entropy Critical Values		
			h^l*0.05^left = 7.9019 h^l*0.05^right = 7.9030	h^l*0.01^left = 7.9017; h^l*0.01^right = 7.9032	h^l*0.001^left = 7.9015l h^l*0.001^right = 7.9034
Lena	7.9978	7.9028	Pass	Pass	Pass
Baboon	7.9977	7.9023	Pass	Pass	Pass
Boat	7.997	7.9027	Pass	Pass	Pass
Couple	7.9974	7.9022	Pass	Pass	Pass

**Table 7:** Histogram statistics with the variance and standard deviation of plain and encrypted images.

**Plain Image**	**Scale**	α	β
Lena 256×256	gray	37,963	195
Reference [22] (lena 256×256)	gray	38,451	196
Boat 256×256	gray	103,380	321.5
Baboon 256×256	gray	58,542	241.9
Couple 256×256	gray	79,457	281.9
**Encrypted Image**	**Scale**		
Lena 256×256	gray	230	15
Reference [22] (lena 256×256)	gray	414	20
Boat 256×256	gray	250	15.8
Baboon 256×256	gray	260	16.1
Couple 256×256	gray	242	15.6

**Table 8:** Test results for the correlation coefficient between plaintext and ciphertext images.

Images	Horizontal Correlation Coefficient		Vertical Correlation Coefficient		Diagonal Direction Correlation Coefficient	
	Clear Image	Ciphertext Image	Clear Image	Ciphertext Image	Clear Image	Ciphertext Image
Lena	0.971	0.012	0.9402	0.002	0.9121	−0.0083
Baboon	0.8343	−0.0109	0.8712	0.0043	0.794	−0.0074
Boat	0.9574	−0.0076	0.9533	−0.0137	0.915	0.0036

## References

[1] Peng Z.P., Wang C.H., Yuan L., Luo X.W. (2014). A novel four-dimensional multi-wing hyper-chaotic attractor and its application in image encryption. Acta Phys. Sin. Chin. Ed..

[2] Liu Y. (2015). Study on Chaos Based Pseudorandom Sequence Algorithm and Image Encryption Technique. Ph.D. Thesis.

[3] Rui L. (2015). New Algorithm for Color Image Encryption Using Improved 1D Logistic Chaotic Map. Open Cybern. Syst. J..

[4] Sun S. (2018). A Novel Hyperchaotic Image Encryption Scheme Based on DNA Encoding, Pixel-Level Scrambling and Bit-Level Scrambling. IEEE Photonics J..

[5] Chai X., Gan Z., Zhang M. (2016). A fast chaos-based image encryption scheme with a novel plain image-related swapping block permutation and block diffusion. Multimed. Tools Appl..

[6] Ahmad J., Khan M.A., Ahmed F., Khan J.S. (2017). A novel image encryption scheme based on orthogonal matrix, skew tent map, and XOR operation. Neural Comput. Appl..

[7] Ahmad J., Hwang S.O. (2015). Chaos-based diffusion for highly autocorrelated data in encryption algorithms. Nonlinear Dyn..

[8] Gao T., Chen Z. (2018). Image encryption based on a new total shuffling algorithm. Chaos Solitons Fractals.

[9] Pareek N.K., Patidar V., Sud K.K. (2006). Image encryption using chaotic logistic map. Image Vis. Comput..

[10] Li Y., Tang W.K.S., Chen G. (2005). Generating hyperchaos via state feedback control. Int. J. Bifurc. Chaos.

[11] Li Y., Wang C., Chen H. (2017). A hyper-chaos-based image encryption algorithm using pixel-level permutation and bit-level permutation. Opt. Lasers Eng..

[12] Zhang X., Feng H., Ying N. (2017). Chaotic image encryption algorithm based on bit permutation and dynamic DNA encoding. Comput. Intell. Neurosci..

[13] Liu J., Yang D., Zhou H., Chen S. (2017). A digital image encryption algorithm based on bit-planes and an improved logistic map. Multimed. Tools Appl..

[14] Qi Y., Wang C. (2018). A New Chaotic Image Encryption Scheme Using Breadth-First Search and Dynamic Diffusion. Int. J. Bifurc. Chaos.

[15] Assad S.E., Farajallah M. (2015). A new chaos-based image encryption system. Signal Process. Image Commun..

[16] Çavuşoğlu Ü., Panahi S., Akgül A., Jafari S., Kaçar S. (2019). A new chaotic system with hidden attractor and its engineering applications: Analog circuit realization and image encryption. Analog Integr. Circuits Signal Process..

[17] Enayatifar R., Abdullah A.H., Isnin I.F., Altameem A., Lee M. (2017). Image encryption using a synchronous permutation-diffusion technique. Opt. Lasers Eng..

[18] Chen J.X., Zhu Z.-L., Fu C., Zhang L.-B., Zhang Y. (2015). An image encryption scheme using nonlinear inter-pixel computing and swapping based permutation approach. Commun. Nonlinear Sci. Numer. Simul..

[19] Zhang Y., Li C., Li Q., Zhang D., Shu S. (2012). Breaking a chaotic image encryption algorithm based on perceptron model. Nonlinear Dyn..

[20] Sprott J. (1994). Some simple chaotic flows. Phys. Rev. E.

[21] Alvarez G., Li S. (2006). Some basic cryptographic requirements for chaos-based cryptosystems. Int. J. Bifurc. Chaos.

[22] Ravichandran D., Praveenkumar P., Rayappan J.B.B., Amirtharajan R. (2017). DNA Chaos Blend to Secure Medical Privacy. IEEE Trans. Nanobiosci..

[23] Murillo-Escobar M.A., Meranza-Castillón M.O., López-Gutiérrez R.M., Cruz-Hernández C. (2019). Suggested Integral Analysis for Chaos-Based Image Cryptosystems. Entropy.

[24] Li S., Ding W., Yin B., Zhang T., Ma Y. (2018). A Novel Delay Linear Coupling Logistics Map Model for Color Image Encryption. Entropy.

